# Supplementary material for: A fault-intrusion-tolerant system and deadline-aware algorithm for scheduling scientific workflow in the cloud
Source: PeerJ Comput Sci. 2021 Nov 2;7:e747. doi: 10.7717/peerj-cs.747 (PMC8576554; doi:10.7717/peerj-cs.747)
Supplement: Supplemental Information 6 [file peerj-cs-07-747-s006.zip › Myproject1.1/docs/org/cloudbus/cloudsim/examples/CloudSimExample8.GlobalBroker.html]

CloudSimExample8.GlobalBroker


JavaScript is disabled on your browser.


- Overview
- Package
- Class
- Use
- Tree
- Deprecated
- Index
- Help

- Prev Class
- Next Class

- Frames
- No Frames

- All Classes

- Summary:
- Nested |
- Field |
- Constr |
- Method

- Detail:
- Field |
- Constr |
- Method


org.cloudbus.cloudsim.examples

## Class CloudSimExample8.GlobalBroker

- java.lang.Object
- - org.cloudbus.cloudsim.core.SimEntity
  - - org.cloudbus.cloudsim.examples.CloudSimExample8.GlobalBroker

- All Implemented Interfaces:
  :   java.lang.Cloneable

  Enclosing class:
  :   CloudSimExample8

  ---

    

  ```
  public static class CloudSimExample8.GlobalBroker
  extends SimEntity
  ```

- - ### Field Summary

    - ### Fields inherited from class org.cloudbus.cloudsim.core.SimEntity

      `FINISHED, HOLDING, RUNNABLE, WAITING`
  - ### Constructor Summary

    Constructors

    Constructor and Description

    `CloudSimExample8.GlobalBroker(java.lang.String name)`
  - ### Method Summary

    Methods

    | Modifier and Type | Method and Description |
    | --- | --- |
    | `DatacenterBroker` | `getBroker()` |
    | `java.util.List<Cloudlet>` | `getCloudletList()` |
    | `java.util.List<Vm>` | `getVmList()` |
    | `void` | `processEvent(SimEvent ev)` This method is invoked by the `Simulation` class whenever there is an event in the deferred queue, which needs to be processed by the entity. |
    | `protected void` | `setBroker(DatacenterBroker broker)` |
    | `protected void` | `setCloudletList(java.util.List<Cloudlet> cloudletList)` |
    | `protected void` | `setVmList(java.util.List<Vm> vmList)` |
    | `void` | `shutdownEntity()` This method is invoked by the `Simulation` before the simulation finishes. |
    | `void` | `startEntity()` This method is invoked by the `Simulation` class when the simulation is started. |

    - ### Methods inherited from class org.cloudbus.cloudsim.core.SimEntity

      `cancelEvent, clone, getEventBuffer, getId, getName, getNextEvent, getNextEvent, getState, numEventsWaiting, numEventsWaiting, pause, run, schedule, schedule, schedule, schedule, scheduleFirst, scheduleFirst, scheduleFirst, scheduleFirst, scheduleFirstNow, scheduleFirstNow, scheduleFirstNow, scheduleFirstNow, scheduleNow, scheduleNow, scheduleNow, scheduleNow, selectEvent, send, send, send, send, sendNow, sendNow, sendNow, sendNow, setEventBuffer, setId, setState, waitForEvent`
    - ### Methods inherited from class java.lang.Object

      `equals, finalize, getClass, hashCode, notify, notifyAll, toString, wait, wait, wait`

- - ### Constructor Detail


    - #### CloudSimExample8.GlobalBroker

      ```
      public CloudSimExample8.GlobalBroker(java.lang.String name)
      ```
  - ### Method Detail


    - #### processEvent

      ```
      public void processEvent(SimEvent ev)
      ```

      **Description copied from class: `SimEntity`**

      This method is invoked by the `Simulation` class whenever there is an event in the
      deferred queue, which needs to be processed by the entity.

      **Specified by:**
      :   `processEvent` in class `SimEntity`

      Parameters:
      :   `ev` - the event to be processed by the entity


    - #### startEntity

      ```
      public void startEntity()
      ```

      **Description copied from class: `SimEntity`**

      This method is invoked by the `Simulation` class when the simulation is started. This
      method should be responsible for starting the entity up.

      **Specified by:**
      :   `startEntity` in class `SimEntity`


    - #### shutdownEntity

      ```
      public void shutdownEntity()
      ```

      **Description copied from class: `SimEntity`**

      This method is invoked by the `Simulation` before the simulation finishes. If you want
      to save data in log files this is the method in which the corresponding code would be placed.

      **Specified by:**
      :   `shutdownEntity` in class `SimEntity`


    - #### getVmList

      ```
      public java.util.List<Vm> getVmList()
      ```


    - #### setVmList

      ```
      protected void setVmList(java.util.List<Vm> vmList)
      ```


    - #### getCloudletList

      ```
      public java.util.List<Cloudlet> getCloudletList()
      ```


    - #### setCloudletList

      ```
      protected void setCloudletList(java.util.List<Cloudlet> cloudletList)
      ```


    - #### getBroker

      ```
      public DatacenterBroker getBroker()
      ```


    - #### setBroker

      ```
      protected void setBroker(DatacenterBroker broker)
      ```


- Overview
- Package
- Class
- Use
- Tree
- Deprecated
- Index
- Help

- Prev Class
- Next Class

- Frames
- No Frames

- All Classes

- Summary:
- Nested |
- Field |
- Constr |
- Method

- Detail:
- Field |
- Constr |
- Method
